# Nodular Worm Infection in Wild Chimpanzees in Western Uganda: A Risk for Human Health?

**DOI:** 10.1371/journal.pntd.0000630

**Published:** 2010-03-16

**Authors:** Sabrina Krief, Benjamin Vermeulen, Sophie Lafosse, John M. Kasenene, Adélaïde Nieguitsila, Madeleine Berthelemy, Monique L'Hostis, Odile Bain, Jacques Guillot

**Affiliations:** 1 UMR 7206-USM 104-Eco-anthropologie et ethnobiologie, Muséum National d'Histoire Naturelle, Paris, France; 2 UMR AFSSA, ENVA, Paris 12, BIPAR, Ecole Nationale Vétérinaire d'Alfort, Maisons-Alfort, France; 3 Makerere University Biological Field Station, Fort Portal, Uganda; 4 Service de Parasitologie, Ecole Nationale Vétérinaire, Agroalimentaire et de l'Alimentation Nantes-Atlantique, Nantes, France; 5 UMR 7205 CNRS, Parasitologie comparée, Muséum National d'Histoire Naturelle, Paris, France; Fundacao Oswaldo Cruz, Brazil

## Abstract

This study focused on *Oeosophagostomum* sp., and more especially on *O. bifurcum*, as a parasite that can be lethal to humans and is widespread among humans and monkeys in endemic regions, but has not yet been documented in apes. Its epidemiology and the role played by non-human primates in its transmission are still poorly understood. *O. stephanostomum* was the only species diagnosed so far in chimpanzees. Until recently, *O. bifurcum* was assumed to have a high zoonotic potential, but recent findings tend to demonstrate that *O. bifurcum* of non-human primates and humans might be genetically distinct. As the closest relative to human beings, and a species living in spatial proximity to humans in the field site studied, *Pan troglodytes* is thus an interesting host to investigate. Recently, a role for chimpanzees in the emergence of HIV and malaria in humans has been documented. In the framework of our long-term health monitoring of wild chimpanzees from Kibale National Park in Western Uganda, we analysed 311 samples of faeces. Coproscopy revealed that high-ranking males are more infected than other individuals. These chimpanzees are also the more frequent crop-raiders. Results from PCR assays conducted on larvae and dried faeces also revealed that *O. stephanostomum* as well as *O. bifurcum* are infecting chimpanzees, both species co-existing in the same individuals. Because contacts between humans and great apes are increasing with ecotourism and forest fragmentation in areas of high population density, this paper emphasizes that the presence of potential zoonotic parasites should be viewed as a major concern for public health. Investigations of the parasite status of people living around the park or working inside as well as sympatric non-human primates should be planned, and further research might reveal this as a promising aspect of efforts to reinforce measures against crop-raiding.

## Author Summary

The disease caused by the nodular worm *Oesophagostomum bifurcum* can be lethal in humans and is thus of major human health significance in certain African regions. There are still gaps in the understanding of the epidemiology of the disease, including the role of non-human primates as reservoirs of the infection. We recently conducted a survey in a community of wild chimpanzees (*Pan troglodytes schweinfurthii*) in Kibale National Park, Western Uganda. *O. stephanostomum* is so far the only species previously found in chimpanzees. A total of 311 stool samples was examined and revealed that high-ranking males are more infected than other individuals. These chimpanzees are also the more frequent crop-raiders. Moreover, we reported for the first time molecular evidence for *O. bifurcum* in addition to *O. stephanostomum* in chimpanzees. Our results raise public health concerns for a neglected infection in regions where spatial proximity between great apes and humans are increasing because of forest fragmentation.

## Introduction

Nodular worms (*Oesophagostomum* spp.) are commonly found as nematode parasites of pigs, ruminants and primates, including humans. In endemic foci in Africa, especially in Ghana and Togo, a high prevalence of *Oesophagostomum bifurcum* infection has been reported in human populations, one million being estimated at risk [1],[2]. Patients are mostly children aged <10 years [2]. Clinical disease, due to encysted larvae, known as oesophagostomosis, sometimes leads to death [1]–[3]. The distinction between hookworm and nodular worms eggs is not possible [2] and the definitive diagnosis of oesophagostomosis in humans involved exploratory surgery or ultrasound examination. Transmission occurs through the ingestion of the infective third-stage larvae (L3) but the factors explaining such a high regional prevalence remain unknown. Eight species of *Oesophagostomum* have been recognized so far to occur in non-human primates [4]. Among them, *O. bifurcum*, *O. stephanostomum* and *O. aculeatum* are also reported in humans [3]. Human cases have been attributed to a zoonotic origin, non-human primates being proposed as a potential reservoir [3]. However experimental infection of rhesus monkey (*Macaca mulata*) showed that *O. bifurcum* obtained from humans did not effectively infect monkeys [5]. In addition, significant variations exist in lengths of adult worms isolated from humans and non-human primates [4]. The geographic distribution in humans and some non-human primates is not overlapping [6],[7] and recent molecular findings demonstrated a genetic host-affiliated sub-structuring within *O. bifurcum* [6],[7]. Among great apes, especially chimpanzees, bonobos and gorillas, prevalence of strongyle eggs in stools is often high and *O. stephanostomum* was the only species of *Oesophagostomum* identified so far [8]–[10]. However, little is known about the intensity of infection in terms of parasite load and clinical signs in great apes. It has been reported that wild apes develop clinical signs of oesophagostomosis as soon as in captivity [11] while the presence of parasites remains asymptomatic in wild animals. Recently fatal cases have been described in African apes from sanctuaries [12] and collected parasites were diagnosed as *O. stephanostomum*. Nevertheless, because of the phylogenetic and spatial proximity between humans and chimpanzees, potential transmission is not excluded especially in Uganda where human oesophagostomosis has been reported [4]. Around Kibale, population density is high (up to 512 ind/km^2^) [13] and chimpanzees regularly crop-raid. Additionally recent findings confirmed that human-related diseases should be considered as a high threat for endangered apes [14]–[18]. As a consequence, it has been emphasized that investigations on potential cross-transmission should be reinforced. We report hereafter the results of our recent finding about nodular worm infection in wild chimpanzees (*Pan troglodytes schweinfurthii*) in the framework of a long-term health monitoring of the community of Kanyawara in Kibale National Park (Uganda).

## Methods

### Study site and study periods

The studied chimpanzees (*Pan troglodytes schweinfurthhii*) belonged to one community in Kibale National Park (766 km^2^, 0°13′–0°41′N, 30°19′–30°32′E), located in Kanyawara area. This community counted 52 chimpanzees in 2006. Ages presented are those estimated in 2006. Their home range is close to the boundary of the Park and Kanyawara chimpanzees are sometimes entering plantations for crop-raiding. Stool samples were collected from identified individuals within the minutes following defecation.

We performed analyses on two series of fecal samples (Table 1). From December 2005 to March 2006, a total of 295 fecal samples was collected from 33 chimpanzees, 17 females (13 adult females and four immature females) and 16 males (9 adult chimpanzees including five dominant individuals, four subordonate individuals and seven immature males) (set 1); coproscopy, coproculture and molecular analysis were performed on the total or parts of this set. In October 2008, 16 samples were collected from 10 identified chimpanzees, 5 females and 5 males. These samples were dried for further molecular analysis (set 2). Indeed, since coprocultures in field conditions and diagnosis of third-stage larvae (L3) are laborious and require skilled personnel for identification, we wished to test a molecular method using dried feces.

**Table 1:** Analyses for *Oesophagostomum* sp. identification from fecal samples of wild chimpanzees collected in two periods.

Method of analysis	Set of samples	Type of fecal samples	Level of identification for strongyloid nematodes			Positive results	
			Super-family (*Strongyloidea*)	Genus (*Oesophagostomum*)	Species	Chimpanzees (n)	Samples (N)
Direct examination	Set 1	formalin samples	X			60% (33)	12% (295)
Mac Master flottation	Set 1	fresh samples	X			100% (29)	91% (100)
Coproculture	Set 1	fresh samples	X	X		77% (13)	75% (16)
PCR-RFLP	Set 1	Copro-cultured samples	X	X	X	23% (13)	19% (16)
Semi nested PCR and sequencing	Set 1	Copro-cultured samples	X	X	X	46% (13)	37% (16)
Direct PCR and sequencing	Set 2	dried samples	X	X	X	100% (10)	87% (16)

**S**et 1: December 2005 to March 2006; set 2: October 2008; n: number of chimpanzees studied, N: number of sampled analysed, X: level of identification possible considering the method and sample used).

### Coproscopy

For each sample of set 1 (n = 295), two grams of fresh stool were preserved in 18 mL of 10% formalin saline solution, then smears made with 50 µL of the suspension were microscopically examined. MacMaster flotation was performed at the field station on fresh stools within the day of collection. MacMaster cells were filled with one mL of filtrat of two grams of fresh stools diluted in 30 mL of magnesium sulfate. However, as electricity was not available every day, only 100 samples could be examined.

With both methods, strongyloid eggs were identified according to their size, color, shape and morula aspect (16–32 cells) and they were counted. Egg per gram (epg) counts were corrected according to the fecal consistency (ie ×2 for soft stools and ×3 for diarrheic stools) [12]. Arithmetic corrected mean was calculated including infected and non infected samples (mean abundance). Larvae of *Probstmayria* sp. and larvae of unidentified species as well as ciliates were also counted during coproscopy (data not shown).

### Coprocultures

To confirm identification of strongyloid eggs in set 1, coprocultures were performed with 16 stool samples from 13 individuals (5 males, 8 females). After 10 to 15 days of incubation, larvae were collected by Baermann technique and preserved in 95% ethanol. Third-stage larvae (L3) of *Oesophagostomum* spp. obtained were microscopically diagnosed (long filamentous tail of the sheath, triangular intestinal cells, and length of the larvae [4]).

### Molecular analysis

Molecular characterization was performed on samples from sets 1 and 2. With the mixture of larvae obtained from each above coproculture (n = 16 samples, set 1), DNA was prepared using Nucleo-spin Tissue (Macherey-Nagel) and ITS2 region was amplified using the primers NC1 and NC2 as described previously [19]. ITS2 sequence of *O. stephanostomum* is characterized by 2 digestion sites for *NLa*III while ITS2 sequence of *O. bifurcum* is characterized by a unique digestion site. RFLP were analyzed after digestion of the ITS2 sequence. Sequencing was performed on ITS2 sequences and compared to published data (GenBank accession numbers: AF136576 for *O. stephanostomum*; AF136575 and Y11733 for *O. bifurcum*). Another PCR test was performed from DNA obtained from the cultured samples to compare the two methods. We used a semi-nested PCR followed by direct sequencing as described before [20].

From set 2, 16 samples of 4 g fresh feces were stored dried on 20g of silicagel bead. Before DNA extraction, vegetal debris was removed in order to avoid PCR inhibition. DNA was extracted from 200mg of dried feces without culture by using the QIAMP DNA Stool Kit (Qiagen, Chatsworth, CA) according to instructions with the following modifications: in step 3, the suspension of 200 mg with buffer ASL was incubated overnight at 70°C and in step 12, the solution was incubated one hour with proteinase K at 70°C. Direct sequencing after PCR using NC1 and NC2 primers was performed.

## Results

Table 1 presents results obtained from the two sets of collection with the different methods of analysis.

### Prevalence of strongyloid infection with egg counting

Strongyloid eggs were detected in 12% of the 295 feces examined with direct smears, that is 60% of the chimpanzees (n = 33). The arithmetic mean corrected parasite load of strongyloid eggs was 52±12 epg. The diarrheic samples had a significantly higher oesophagostomine egg counts (225 epg, n = 17) than the firm feces (19 epg, n = 217) (Kruskal-Wallis test: P<0.01). No other factor was significantly affecting egg counts by direct examination although egg counts tend to be affected by hierarchical status in males (dominants: 42 epg, n = 33, subordinates: epg: 14, n = 42).

Strongyloid eggs were detected at least once from all the chimpanzees (n = 29) with MacMaster method. The proportion of positive samples for strongyloid eggs with Mac Master flotation was 91%. The arithmetic mean corrected parasite load of strongyloids was 140±58 epg. Values of corrected epg were significantly different according to hierarchical status in males (dominants: 232 epg, n = 10, subordonates: 88 epg, n = 13; Mann-Whitney test; P value = 0.005) and fecal consistency (firm feces: 90 epg, n = 73; soft feces: 239 epg, n = 20; diarrheic feces: 414 epg, n = 7; Kruskal-Wallis test: P value = 0.021). No difference according to the sex, the age or the sampling period of the day was found.

### Prevalence of *Oesophagostomum* spp. infection

L3 characteristic of *Oesophagostomum* were found after coproculture and microscopic examination in 12/16 samples from 10/13 chimpanzees (3 males; 10 females) of the set 1.

### Species identification with molecular analysis

**Figure 1:**
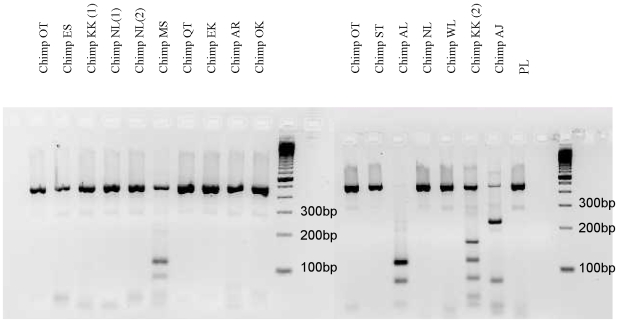
PCR-RFLP of ITS2 using the endonuclease *Nla*III of DNA samples of *Oesophagostomum stephanostomum* (chimpanzees MS and AL) and *Oesophagostomum bifurcum* (chimpanzee AJ) extracted from coprocultured feces of 13 wild chimpanzees. The 13 chimpanzees sampled are: AJ, male, 31 yr old; AL, female, 23 yr old; BB, male, 39 yr old; KK, male, 20 yrs-old; MS male 26 yr old; OG, male, 4 yr old; OM, female 1 yr old; OK, female, 11 yr old; OU, female, 26 yr old; OT, female, 7 yr old; PG, male 17 yr old; ST, male, 50 yr old; WL, female, 13 yr old. DNA from 16 samples of these chimpanzees was extracted and digested. 2 different samples from NL and from KK were analysed, one sample from OK has been tested twice. PCR products at 500 bp correspond to DNA from *Panagrolaimus* sp. and PCR product of the sample KK (2) to *Necator* -like DNA species. Control with *Panagrolaimus* larva (PL).

**Figure 2:**
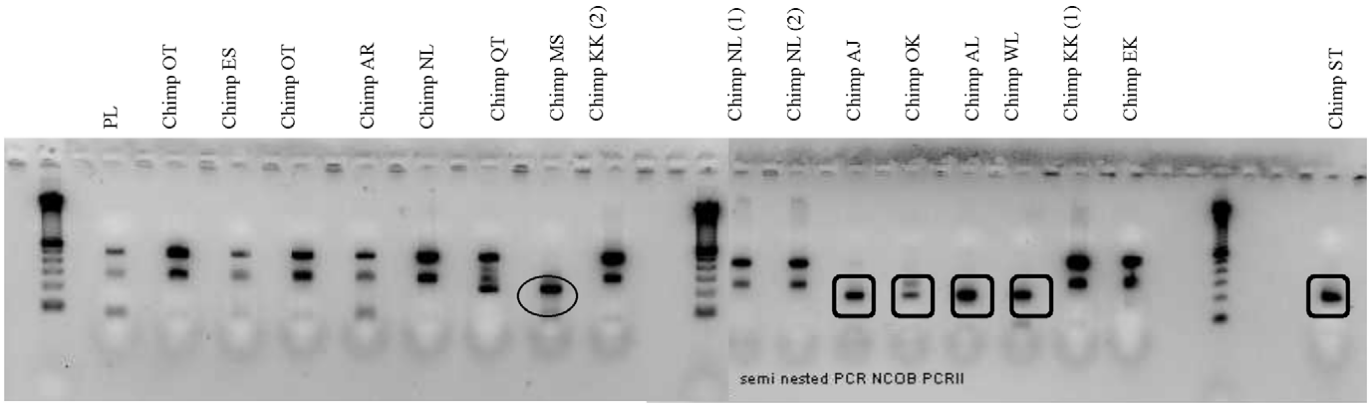
Semi-nested PCR and direct sequencing on DNA extracted from 16 coprocultured samples of 13 wild chimpanzees (see identity in Figure 1). Two different samples from NL and from KK were analysed, one sample from OK has been tested twice. Circle: *Oesophagostomum stephanostomum* products sequenced; square: *Oesophagostomum bifurcum* products sequenced. Control with *Panagrolaimus* larvae (PL).

PCR-RFLP conducted on larvae from the 16 coprocultured samples (set 1) from wild chimpanzees identified *O. stephanostomum* and *O. bifurcum*. ITS2 sequence of *O. stephanostomum*, characterized by 2 digestion sites for *NLa*III, was identified from fecal samples from 2 chimpanzees (MS, male and AL, female). ITS2 sequence of *O. bifurcum*, characterized by a unique digestion site, was identified from one fecal sample from one chimpanzee (AJ, male) (Fig. 1). Sequencing performed on these samples confirmed the presence of the two species. All but one samples revealed DNA sequences showing 99% of homology with *Panagrolaimus* sp. (AY878405 from Genbank) and one sample revealed 82–88% homology with *Necator* sp. (AF217891 from Genbank) nematodes. With semi-nested PCR and direct sequencing, *O. stephanostomum* was identified in one of the two chimpanzees positive with PCR-RFLP (MS, male) and *O. bifurcum* in five chimpanzees (Fig. 2). In the second set of fecal samples, which were stored dried, *Oesophagostomum* DNA was found in 14 of the 16 fecal samples. All the 10 chimpanzees sampled in set 2 were positive.

All the sequences obtained from extraction of larvae DNA and dried feces were identical between them for each of the two *Oesophagostomum* species. The sequences corresponding to *O. stephanostomum* were 100% identical to the ITS2 reference sequence of *O. stephanostomum* collected from a chimpanzee in Tanzania (GenBank accession number AF136576) (BLASTn). In the sequences we obtained there was no mixed sequence signals in positions 116, 176, 197 in which the reference sequence shows polymorphism. (fig. 2). Among the three sites where polymorphism is observed in ITS-2 sequences for human and monkeys for *O. bifurcum* (positions 56, 112, 162), there is no nucleotides equivoque in our sequences. Our sequences were identical to the ITS2 sequence of *O. bifurcum* collected from a human (100% of identity with GenBank accession number Y11733) and different in position 112 from the one documented in *Cercopithecus mona* (GenBank accession number AF136575). The species *O. stephanostomum* was found in 11 chimpanzees. The species *O. bifurcum* was found in 5 chimpanzees. One chimpanzee was co-infected by two *Oesophagostomum* species in the first period (AL, female) and four chimpanzees (2 females AJ and WL and two males AL and ST) were infected by the two species if we considered both periods (December 2005 to March 2006 and October 2008). Whatever the method used to identify specimens of the genus *Oesophagostomum* (coproculture or any molecular characterization), a total number of 26 of 32 samples (81%) were positive corresponding to 15 chimpanzees of the 18 sampled (83%).

## Discussion

In this study we used several methods to survey parasite status of wild chimpanzees.

We compared nodular worm eggs counts between individuals of different classes of age, sex and dominance rank. We demonstrated for the first time that wild chimpanzees could be infected by *O. bifurcum*. The presence of two *Oesophagostomum* species (*O. stephanostomum* and *O. bifurcum*) was reported in the same chimpanzees community. Our results, based on RFLP-PCR and semi-nested PCR-direct sequencing and PCR from dried stools, extend our understanding of the epidemiology of *O. bifurcum*, confirm accuracy of alternative method (DNA extraction from dried stools) to coproculture and raise public health awareness for a neglected disease.

However while the substantial increase of accuracy of PCR compared to coproscopy has been previously shown [21], technical difficulties and limitations of stool analyses and culture due to field conditions when studying wild chimpanzees have to be considered. They are overcome by using PCR directly on dried stools. Additionally, the sensitivity of molecular analysis was higher when applied directly on dried samples than samples obtained from coproculture. With both methods of coproscopy, we determined that high-ranking males in Kanyawara chimpanzee community had higher parasite burdens during the study period. Our results also provided evidence that these free-ranging chimpanzees are infected by two *Oesophagostomum* species (*O. stephanostomum* and *O. bifurcum*). The species *O. bifurcum* is responsible for human and monkeys infections and had never been characterized in wild great apes as chimpanzees so far. The species *O. stephanostomum* is detected in great apes and this species was recently associated with nodular lesions in chimpanzees and a gorilla from sanctuaries [12]. Behavioral patterns of *Pan troglodytes* may explain that males are more infected than females by strongyloid parasites: male chimpanzees are staying all their life in their native community while females migrate. Males develop close relationships, indulging in very long grooming sessions where individuals are staying in close proximity. Our results are consistent with previous studies showing that both testosterone and cortisol were positively associated with gastrointestinal parasite infections in Kibale chimpanzees [22] suggesting that stress of high-ranking males may alter an efficient immune response. Additionally males are visiting plantations in the edge of the forest more frequently than females, encountering conditions favoring parasite transmission from humans and non-human primates: people being very close to the forest are usually not using latrines and monkeys in the edge such as red colobus are more infected than those from the interior, egg counts for *Oesophagostomum* being 10 times higher [23].

In spite of the severe health problem caused by oesophagostomosis to humans, epidemiology and transmission of the disease are still poorly understood [24]. While colobus monkeys were not infected in surveys conducted in Ghana [20],[21], in Kibale NP, primates including the arboreal red colobus (*Piliocolobus tephrosceles*) and black and white colobus (*Colobus guereza*) and more terrestrial species such as olive baboons (*Papio anubis*) were proved to be infected by *Oesophagostomum* sp. [23],[25],[26]. Diagnosis of the parasites species was not conducted in monkeys but previous findings suggesting no risk of infection for arboreal colobus monkeys [24] was not supported at the genus level in this area. The role of chimpanzees and other primates in the cycle needs thus to be further explored. *Oesophagostomum bifurcum* nematodes from chimpanzees may be genetically distinct from *O. bifurcum* nematodes from other primate species including humans as previously demonstrated [6],[7]. However, chimpanzees are more closely related to humans than non-human primates species investigated so far (colobus, baboons, patas and Mona monkeys) and investigating the genetic variability of *O. bifurcum* between chimpanzees and other primates would be interesting. Moreover, the home ranges of chimpanzees from Kibale NP include areas where human beings are present. Chimpanzees are visiting plantations bordering their forest home range and males, especially high-ranking males, which have higher infection level, more frequently. Humans are working or entering inside the park (researchers, research assistants, other employees from the park, poachers…). Chimpanzees are also exploiting resources also used by other non-human primates. For these reasons, even if the origin of infection is unknown, the zoonosis risk can not be excluded. Outbreaks of oesophagostomosis in human population have not been documented in the study area. However, an investigation of the parasite status in humans living in the villages surrounding the park should be planed. The presence of potentially zoonotic parasites in chimpanzees in a context where proximity between human and apes is increasing (ecotourism, crop-raiding, research…) should be viewed as a point of concern for the future of public health in this region and elsewhere. For economic and health reasons, prevention of crop-raiding programs should be reinforced.

## Supporting Information

Alternative Language Abstract S1 French translation of the abstract by SK.
(0.03 MB DOC)
