# Supplementary material for: Nodular Worm Infection in Wild Chimpanzees in Western Uganda: A Risk for Human Health?
Source: PLoS Negl Trop Dis. 2010 Mar 16;4(3):e630. doi: 10.1371/journal.pntd.0000630 (PMC2838776; doi:10.1371/journal.pntd.0000630)
Supplement: Alternative Language Abstract S1 — French translation of the abstract by SK. (0.03 MB DOC) [file pntd.0000630.s001.doc]

**Résumé en français**

La maladie causée par le parasite nodulaire *Oesophagostomum bifurcum* peut être létale chez l’homme et présente donc un risque majeur dans certaines régions d’Afrique. L’épidémiologie de la maladie est encore mal comprise en particulier le rôle des primates comme réservoir de l’infection. Nous avons mené récemment une étude dans la communauté de chimpanzés sauvages (*Pan troglodytes schweinfurthii*) du parc national de Kibale dans l’Ouest de l’Ouganda. 311 échantillons de selles ont été analysés et ont révélé que les mâles dominants sont plus fréquemment infectés que les autres individus. Ces chimpanzés sont ceux qui pénètrent le plus fréquemment les cultures des villageois pour les piller. De plus, notre étude moléculaire a mis en évidence pour la première fois chez les chimpanzés la présence d’*O. bifurcum* en plus de celle d’*O. stephanostomum*. Nos résultats soulignent l’importance de prendre en compte cette maladie souvent négligée dans des régions où la proximité spatiale entre les grands singes et les hommes s’accroît à cause de la déforestation.
